# Subliminal Affect Valence Words Change Conscious Mood Potency but Not Valence: Is This Evidence for Unconscious Valence Affect?

**DOI:** 10.3390/brainsci2040504

**Published:** 2012-10-17

**Authors:** Howard Shevrin, Jaak Panksepp, Linda A. W. Brakel, Michael Snodgrass

**Affiliations:** 1 Department of Psychiatry, University of Michigan Medical Center, 4250 Plymouth Rd., Ann Arbor, MI 48109, USA; Email: brakel@umich.edu (L.A.W.B.); jmsnodgr@umich.edu (M.S.); 2 Department of Veterinary and Comparative Anatomy, Pharmacology, and Physiology, Washington State University, PO Box 646520, Pullman, WA 99164, USA; Email: jpanksepp@vetmed.wsu.edu

**Keywords:** affect, emotion, conscious, unconscious, subliminal, objective threshold, subjective threshold

## Abstract

Whether or not affect can be unconscious remains controversial. Research claiming to demonstrate unconscious affect fails to establish clearly unconscious stimulus conditions. The few investigations that have established unconscious conditions fail to rule out conscious affect changes. We report two studies in which unconscious stimulus conditions were met and conscious mood changes measured. The subliminal stimuli were positive and negative affect words presented at the objective detection threshold; conscious mood changes were measured with standard manikin valence, potency, and arousal scales. We found and replicated that unconscious emotional stimuli produced conscious mood changes on the potency scale but not on the valence scale. Were positive and negative affects aroused unconsciously, but reflected consciously in potency changes? Or were the valence words unconscious cognitive causes of conscious mood changes being activated without unconscious affect? A thought experiment is offered as a way to resolve this dilemma.

## 1. Introduction

While considerable evidence has accumulated supporting the existence of unconscious perception and memory [1,2,3,4,5,6], the existence of unconscious affect remains controversial. The controversy centers on theoretical issues and also involves significant methodological concerns. Historically, two major figures in psychology, Freud [7] and James [8], have taken the position that affect can only be conscious. Both defined affect as quintessentially a qualitative conscious experience caused by bodily somatic physiological processes.

More recently, a number of investigators have taken the position that an affect is simply another form of information so that what the affect is *about* may be unconscious, but the emotion itself is a conscious experience [9]. Lambie and Marcel [10] also place great emphasis on the phenomenology of emotion experience, taking the view that what is nonconscious are the processes and representations that underlie the actual conscious experience of the emotion. Interestingly, Zajonc’s [11,12] position, although differing from Lambie and Marcel’s in a number of significant ways, is similar in that what is unconscious causes the conscious emotion experience, but is not itself to be thought of as an emotion. This position finds some support in split brain patients who demonstrate the appropriate affect associated with a stimulus presented to the right hemisphere (e.g., the word “laugh” elicits laughter) but are unable to identify its cause. Breuer and Freud [13] described a similar condition in patients with intact brains but who suffered from hysterical dissociation. This kind of disconnection between expressed and experienced affect without awareness of its cause has also been described in normal hypnotized subjects who enact hypnotic suggestions. In all these instances, however, the instigating stimuli were supraliminal, but were unavailable either by reason of brain damage (split brain patients), psychopathology (hysterical dissociation or repression), changed states of consciousness (hypnosis), leaving open the question as to what happens when the stimuli are entirely unconscious, the subject of interest in the studies to be reported.

Among neuroscientists, Panksepp argues in favor of the conscious nature of affects on quite other grounds. For Panksepp, affects must be qualitatively distinguished from cognitive processes that are capable of becoming unconscious because affects function in an entirely different manner. Cognitive processes provide information about the outer and inner world, but affects provide the internal value codes (e.g., good/bad) necessary to sustain ongoing behavior and could not perform that role if they were unconscious [14,15]. According to Panksepp, affects are generated deep in the brainstem and are intrinsically conscious, while cognitive processes are more closely associated with the cortex and can become unconscious. Neuropeptidergic neuromodulaters regulate distinct affect states, while cognitive processes depend more on informationally resolved glutamatergic transmission. Consciousness rather than being restricted just to higher mental functions is assumed also to be present in the deeper, older parts of the brain [16,17]. On the other hand, Damasio [18] and Berridge and Winkielman [19], among others, although granting all the qualitative differences cited by Panksepp, have concluded that these deeper brain structures subserving “core” affects, are largely implicit and unconscious, and that consciousness emerges when they interact with higher cortical functions. On the basis of SCR, EMG, and EEG evoked potential evidence elicited by subliminal valence stimuli, Shevrin [20] has also taken the position that affects can be unconscious as well as conscious.

The first and second author of this paper are thus on opposite sides of the issue of whether affects can be unconscious. It was this difference emerging from intensive discussions that led to a productive adversarial collaboration resulting in the experiments to be reported. The study was conducted in the Ormond and Hazel Hunt Laboratory at the University of Michigan Medical Center under the first author’s direction. The third and fourth authors are colleagues and collaborators of the first author. They participated in setting the problem, designing the experiments, analyzing the data and contributing to writing the paper. They share the first author’s position on unconscious affect.

The question we address can be stated as follows:

*Is* *it possible to establish that an unconscious affect exists which meets the most exacting subliminal conditions so that there can be no reasonable doubt that the process is unconscious, and furthermore that the unconscious stimulus does not cause a change in conscious mood identical to the content of the subliminal stimulus (e.g., the word “laugh” to the right hemisphere results in laughter) in the absence of any awareness of the subliminal stimulus as the cause?*

As we next turn to examining the relevant literature we will measure success by these two criteria: (1) meeting exacting subliminal conditions, (2) absence of a conscious experience similar to the presumed unconscious cause.

### 1.1. Relevant Studies

From an empirical standpoint, a number of studies have appeared in recent years reporting the existence of unconscious affect (for reviews see [19,21]). However, a careful examination of the methodology in these and other studies reveals great differences in how subliminality was achieved and how the unconscious status of the affect was determined. In view of the broad theoretical divide over whether affects can be unconscious, these differences are crucial in evaluating the validity of the evidence for truly unconscious affect.

### 1.2. Problems in Subliminal Methodology: Differences between Objective and Subjective Thresholds

There are two fundamental ways to operationalize whether unconscious effects exist when employing the method of subliminal activation: (1) Objective threshold approaches, and (2) Subjective threshold approaches. In the former, more conservative approach, stimulus conditions are arranged such that participants cannot discriminate the relevant stimuli above chance in direct forced-choice tasks. In the latter approach, stimulus conditions are arranged such that participants simply deny being aware of the stimuli, even though they can nonetheless discriminate the stimuli above chance. Accordingly, objective threshold conditions are more stringent than subjective threshold conditions [5].

Each approach has potential advantages and disadvantages. As Reingold and Merikle [22] suggested, objective threshold conditions may inadvertently eliminate not only conscious but unconscious influences as well, thus throwing out the unconscious baby with the conscious bathwater. However, reviews of the literature (see [23]) suggest that robust effects are readily obtainable under objective threshold conditions—particularly under objective *detection* threshold (ODT) conditions, widely agreed to be the most stringent conditions of all for establishing that a stimulus is unconscious [24,25]. At the ODT, participants cannot discriminate the sheer presence (*vs.* absence) of a stimulus at all.

On the other hand, as Macmillan and Creelman [25] have noted, basing their critique on signal detection theory, subjective threshold approaches may simply index very faint conscious perceptions that are below participants’ response criterion—that is, so weak that participants possess insufficient confidence to say that they really saw the stimuli. This is a serious problem, which most investigators attempt to surmount by demonstrating convergent qualitative differences between effects obtained under subjective threshold *versus* fully conscious conditions. Even then, however, such qualitative differences may simply differentiate effects produced by weak *versus* strong conscious stimuli [26,27], rather than demonstrate truly unconscious effects.

Nevertheless, subjective threshold methods may capture an important class of genuinely unconscious phenomena—namely, reflectively unconscious events that are phenomenally conscious [28]. The cocktail party experience is a commonplace example of phenomenal consciousness occurring in the absence of reflective consciousness. One hears and yet does not know one has heard a remark made in a nearby exchange; a moment later when attention is directed to what one has just heard, the content of the remark enters reflective consciousness. At that point one is able to recall that in fact one was aware of the remark at that earlier time, but because attention was directed elsewhere the response criteria for consciousness had not been met. In contrast, objective threshold methods index phenomenally unconscious events. Accordingly, a simple conscious/unconscious dichotomy is insufficient. Rather, a tripartite model emerges: (1) Completely unconscious (*i.e.*, both phenomenally and reflectively unconscious); (2) Reflectively unconscious but phenomenally conscious; and (3) Completely conscious (*i.e.*, both phenomenally and reflectively conscious). To ensure that we are dealing with phenomenally unconscious affect it thus becomes critical that the stimuli involved should be at the objective detection threshold.

We have selected two studies that come closest to meeting the two criteria, but each for different reasons fall short: Bernat, Bunce, and Shevrin [29] and Winkielman, Berridge, and Willbarger [30]. In the Bernat *et al.* study in which highly pleasant or unpleasant words according to the Osgood valence criteria [31] were presented, subliminal exposure conditions were at the objective detection threshold (1 ms with luminance at 5 foot/lamberts). Detection *d′* was not significantly different from zero. The main finding was significantly greater positive ERP amplitude across a number of components, particularly strong on the left side for *negative* valence stimuli. Supraliminally the same positive amplitude difference was found, but the effects were largely bilateral. It was possible to deliver a reliable stimulus at 1 ms, not attainable with computer screens, because an electronically triggered tachistoscope was used that made such brief flashes possible.

Although this result would seem to support the existence of unconscious affect at the most stringent threshold conditions, it could be argued by those who favor the affects-must-be-conscious view that it remained possible for a change in *conscious* mood to have occurred consonant with the valence of the subliminal stimuli, even though participants might have remained unaware of its cause. Essentially, this is the position of Zajonc [12] and Ohman, Flykt, and Lundqvist [21], who have published evidence for unconscious emotional stimuli *causing* conscious affect changes, while denying that such stimuli cause unconscious affects. Thus the main limitation of the Bernat *et al.* [29] study is that no effort was made to measure conscious mood changes.

This limitation was not present in two experiments reported by Winkielman, Berridge, and Wilbarger [30] and the experimental method summarized in Berridge and Winkielman [19]. Conscious mood was measured following the exposure of angry, happy, and neutral faces in a computerized forward and backward masking procedure. The participant’s task was to identify the gender of a supraliminal neutral face, used as the backward mask, presented immediately following the subliminal stimulus. Participants varied in their degree of thirst, the second factor in the experiment. The main result of interest was an interaction between subliminal stimulus valence and amount of a drink consumed. Following the happy face, more drink was consumed than following the angry face by those participants who rated themselves as very thirsty at the start of the experiment. The second result of interest was the absence of any rated effect on conscious mood in response to the two classes of emotional stimuli. Citing evidence from other studies that expressive faces are known to cause affect experiences, the authors inferred from their two findings that unconscious affect experience caused changes in drinking consonant with stimulus valence, while causing no concomitant change in conscious affect experience. Therefore, they were prepared to make the strong claim that affects can be unconscious. 

Of critical importance is the claim made by Winkielman *et al.* [30] that on a test of perceptibility performance did not significantly exceed chance. The test only briefly described in the paper was the same procedure as in Winkielman *et al.* [32]. In that report, participants were asked to choose which of two faces had been the one just presented and masked (angry or happy). One of the choices exactly matched the just-presented face; the other was another face differing either in gender or ethnicity, but had the *same* facial expression—that is, the response choices were always both happy or both angry. Results were at chance when these comparisons were made. At first glance the results would appear to have met objective threshold conditions for an identification task rather than a detection task.

However, a close examination of the subliminal conditions in the two experiments reported raise serious doubts concerning how unconscious the stimuli actually were with respect to the relevant subliminal dimension *facial expression*. Neither gender nor ethnicity was at issue. The crucial difference between the faces—whether the expression was angry or happy—was *held constant*; only gender or ethnicity were varied. Accordingly, it is not clear that they achieved the objective identification threshold with *respect to the relevant stimulus dimension*, *facial expression*. Indeed, when Murphy and Zajonc [33] had participants make *this* discrimination (*i.e.*, where the response choices varied in expression), using stimulus exposure conditions likely as or more stringent than Winkielman *et al.* [30], they found that performance was clearly well above chance. With this in mind, had Winkielman *et al.* [30] tested perceptibility for the relevant facial expression dimension they would not have attained the objective but rather subjective threshold conditions. Accordingly, although such effects may safely be regarded as reflectively unconscious, there is little reason to regard them as phenomenally unconscious. If so, their interpretation changes crucially—rather than being completely out of awareness, the subliminal faces were phenomenally conscious but weak, such that participants denied their existence. Further, although still noteworthy, if reflectively unconscious effects were what the authors intended to demonstrate, these effects are clearly less pertinent to the fundamental question of whether emotions can be completely (phenomenally) unconscious. Lastly, the absence of a correlation between perceptibility and conscious mood reported by Winkielman *et al.* [30] is irrelevant because they were not correlating the truly relevant dimension, perceptibility of the difference in facial expression. Also, since there is no clear theory of how affective consciousness may operate, it would have been useful if there had been a psychological measure of thirst or appetite for the drink prior to the drinking test (*i.e.*, the most relevant experienced affect for the behavioral outcome measure was not monitored).

Although in the Bernat *et al.* [29] study stimuli were presented at the objective detection threshold and produced positive results, it failed to incorporate a measure of conscious mood. The Winkielman *et al.* study [30] and the Berridge and Winkielman [19] study, on the other hand, did measure a large variety of conscious mood variables, but failed to establish that the subliminal stimuli were phenomenally unconscious. The need is for a study that both presents emotional stimuli at the objective detection threshold *and* measures conscious mood changes. The study to be reported below undertook to do both. Briefly, in two experiments, two rigorously subliminal blocks of either pleasant or unpleasant words were presented, followed by two blocks of the other valence. During these blocks, participants made dummy bright *vs.* dim ratings on each trial (in actuality, this was not manipulated). Participants rated their conscious mood (on valence, arousal, and potency scales) after each individual block.

## 2. Results and Discussion

Two results of interest were found in the first experiment. They occurred only following a *change* in stimulus valence—that is, when affect ratings of the second *vs.* third blocks were examined (see details of procedure below). First, there were no valence or arousal mood effects—that is, subliminal word valence did *not* affect these conscious moods. Second and critically, subliminal word valence nonetheless *did* cause changes in conscious mood potency. That is, when a shift from either pleasant to unpleasant subliminal words or the reverse occurred, participants’ mood following this shift (*i.e.*, block 3) was either more potent (if stimulus valence had changed from unpleasant to pleasant) or less potent (if stimulus valence had changed from pleasant to unpleasant) than after the preceding, opposite valence block (block 2). In this way, the critical potency mood finding depended not only on subliminal word valence but the *context* of such word valence, occurring only following *changes* in valence. Given the novelty of this finding, we felt it important to attempt replication. Notably, both the absence of valence or arousal mood effects and the change-dependent potency effect were replicated in the second study. See Table 1.

**Table 1:** Visual Analog Scale mood means in centimeters for potency, valence, and arousal scales for the second and third stimulus blocks.

Potency					
**Experiment 1**			**Experiment 2**		
	**Mean**	**SE**		**Mean**	**SE**
Pleasant Stimuli	8.157	0.487	Pleasant Stimuli	7.208	0.469
Unpleasant Stimuli	7.180	0.559	Unpleasant Stimuli	6.471	0.439
*F*(1, 25) = 4.311, *p* = 0.048			*F*(1, 26) = 5.72, *p* = 0.02		
Note: Higher scores are more potent					
**Valence**					
**Experiment 1**			**Experiment 2**		
	**Mean**	**SE**		**Mean**	**SE**
Pleasant Stimuli	6.585	0.555	Pleasant Stimuli	6.355	0.407
Unpleasant Stimuli	6.417	0.455	Unpleasant Stimuli	6.005	0.456
*F*(1, 25) = 0.16, *p* = 0.70			*F*(1, 26) = 0.800, *p* = 0.38		
Note: Higher scores are less pleasant					
**Arousal**					
**Experiment 1**			**Experiment 2**		
	**Mean**	**SE**		**Mean**	**SE**
Pleasant Stimuli	9.132	0.584	Pleasant Stimuli	10.182	0.507
Unpleasant Stimuli	9.405	0.570	Unpleasant Stimuli	10.492	0.454
*F*(1, 25) = 0.63, *p* = 0.44			*F*(1, 26) = 0.548, *p =* 0.466		
Note: Higher scores are less aroused					

Before discussing the potency effect further, additional insight into its (stimulus valence) change-dependent nature is gained by examining the means for all four blocks separately by valence order. See Table 2 (collapsed across both experiments). When analyzed in this way, a Block × Valence Order interaction emerged [*F*(3, 51) = 3.2, *p* = 0.03], carried entirely by a cubic trend: *F*(1, 53) = 9.68, *p* = 0.003 (All Data). This cubic interaction was significant for each experiment separately: *F*(1, 25) = 4.98, *p* = 0.035 (Experiment 1); *F*(1, 26) = 4.81, *p* = 0.037 (Experiment 2), suggesting it is reliable. As Table 2 indicates, when pleasant stimuli were first, potency mood seems to rise from the first to second block (both pleasant), drops significantly after the third (unpleasant) block, and then rises again after the fourth (also unpleasant) block. Analogously, when unpleasant stimuli were first, although here potency mood remains unchanged from the first to second block (both unpleasant), it significantly rises after the third (pleasant) block, but declines after the fourth (also pleasant) block. Overall, this pattern suggests (stimulus valence) change-dependent mood potency effects, followed by adaptation/recovery from this effect.

**Table 2:** Visual Analog Scale mood means in centimeters for potency, valence, and arousal scales for all four stimulus blocks.

Potency				
	**Block 1**	**Block 2**	**Block 3**	**Block 4**
Valence Order				
Pleasant First	6.98 (0.49)	7.75 (0.53)	6.56 (0.52)	7.32 (0.50)
Unpleasant First	7.05 (0.43)	7.06 (0.47)	7.60 (0.46)	6.80 (0.44)
**Valence**				
	**Block 1**	**Block 2**	**Block 3**	**Block 4**
(Across Both Orders)	5.61 (0.31)	6.12 (0.35)	6.54 (0.33)	5.70 (0.38)
**Arousal**				
	**Block 1**	**Block 2**	**Block 3**	**Block 4**
(Across Both Orders)	10.37 (0.32)	9.97 (0.36)	9.65 (0.39)	9.61 (0.43)

Note: Higher scores are more potent but less pleasant or aroused. Standard errors in parentheses.

On the other hand, there were no Block × Valence Order interactions for either mood valence [*F*(3, 51) = 0.43, *ns*] or mood arousal [*F*(3, 51) = 0.95, *ns*], again suggesting that stimulus valence had no effect on conscious valence or arousal mood. See Table 2 for their means by block collapsed across valence order. Further, while valence mood exhibited a Block main effect [*F*(3, 51) = 3.1, *p* = 0.035; carried by a quadratic trend, *F*(1, 53) = 8.5, *p* = 0.005], this was not reliable across experiments [*F*(1, 25) = 10.11, *p* = 0.004 (Experiment 1); *F*(1, 26) = 0.37, *ns* (Experiment 2)]. If this effect is genuine, it reflects a stimulus-independent increase in unpleasant mood until after the third block (perhaps reflecting growing participant boredom, frustration, *etc.*), which then diminishes after the fourth block, perhaps reflecting relief that the experiment is over. Arousal, however, exhibited no Block main effect [*F*(3, 51) = 1.73, *p* = 0.17]. 

This surprising change-dependent potency effect led us to look into what is known about the relationship between the valence and potency scales as measured by the manikin scales. Bradley and Lang [34] have shown that bipolar adjectives drawn from the semantic differential literature distinguish statistically between valence and potency on the two manikin scales. For example, the Submissive-Dominant bipolar adjectives correlate 0.195 with the manikin valence factor and −0.695 with the manikin potency factor; the reverse is true for the Unhappy-Happy bipolar pair, strongly suggesting that valence and potency are in fact largely independent components of affect experience. However, in the same study, Bradley and Lang report a surprisingly high *positive * correlation, 0.79, between these two supposedly orthogonal factors. It is thus possible that our potency effect is really a valence effect in disguise, although this is unlikely given our lack of valence mood effects and the relatively low correlation between these ratings in our data (*r* = −0.24, *p* = 0.08). Nonetheless, in order to take this possibility into account, we adjusted the potency effect for valence (in the same two critical blocks) to see if it would survive. Notably, this adjusted potency effect remained significant: *F*(1, 24) = 4.99, *p* = 0.035 (Experiment 1); *F*(1, 25) = 6.06, *p* = 0.02 (Experiment 2), showing that it is indeed independent from valence.

Baseline means for mood potency, valence, and arousal were 7.55 (SE = 0.35), 4.18 (0.30), and 10.56 (0.42), respectively. When compared to average mood ratings during the experiment (collapse across blocks), participants’ mood potency remained the same [decreasing slightly to 7.14; *t*(54) = 1.36, *p* = 0.18], mood valence became more unpleasant [increasing to 6.01; *t*(54) = −6.46, *p* < 0.001], and tended to become less aroused [decreasing to 9.89; *t*(54) = 1.77, *p* = 0.08]. As would be expected, baseline and average experimental mood was highly positively correlated, *r*(54)s = 0.56, 0.51, and 0.48 (all *p*s < 0.001) for potency, valence, and arousal, respectively. However, baseline mood did not predict any experimental effects (all *p*s > 0.13).

In both experiments, detection *d′* did not differ from zero. In the first, *d′* = 0.01, *t*(26) = 0.13, *p* = 0.9; in the second, *d′* = 0.05. *t*(27) = 1.02, *p* = 0.32. Both experiments were thus at the objective detection threshold, and the critical mood potency effects cannot be attributed to any conscious awareness of the subliminal stimuli. Lastly, bright *vs.* dim stimulus judgments (the dummy task) were unrelated to the subliminal stimuli or the mood ratings.

### 2.1. Discussion

Our findings raise four questions: (1) Why do we find a conscious *potency* mood effect in response to the subliminal valence stimuli rather than a straightforward effect on conscious *valence* mood? (2) Why does this potency effect occur only after a *change* in subliminal stimulus valence? (3) What implications do these findings have for the existence of unconscious affect? and more generally, (4) How can unconscious affect be inferred?

#### 2.1.1. Why a Potency Rather than Valence Conscious Mood Effect?

An interaction between conscious and unconscious processing must be occurring, rather than consciousness simply mirroring what is going on unconsciously. The model for what is happening might be provided by the most common form of interaction between conscious and unconscious processes: priming. In priming, a subliminal stimulus influences the conscious response to a related stimulus. When a word such as *cat* is presented subliminally it will decrease reaction time to *dog* relative to an unrelated word such as *doctor*. One need only replace the target word with the current mood or affect state of the participant.

The priming model, however, runs into difficulty because as mentioned earlier the potency and valence scale are *not* related to each other. Moreover, in priming when the target and prime words are the same or similar, reaction time is fastest. But in the current results participants do not select the option of increasing their ratings on the valence scale when they have the chance to do so, but on the potency scale. The puzzle persists. There appears to be something else happening with subliminally activated affects.

There is another position concerning the relationship between subliminal affect activation and conscious emotional experience. For Zajonc [11] the affect itself is always conscious, but the *causation* may be unconscious. A person might experience free-floating anxiety consciously while remaining unaware of its true cause. The conscious affect thus caused might be assigned to some other target, like a Chinese ideogram as in Murphy and Zajonc [33] or the person’s own mood as in Kunst-Wilson and Zajonc [35], much as in the priming model. But there is a problem with applying this explanation to our results. In the Chinese ideogram study, the affect assigned to the ideogram was in keeping with the valence of the subliminal stimuli. In our study, the subliminal cause was assigned to a different affect dimension even when the participants could have rated themselves as feeling more positive or negative. Instead their moods changed along a potency dimension. 

It could also be argued that valence, potency, and arousal are the three widely recognized dimensions of any affect. There simply was a shift from one dimension of affect to another. This explanation runs into the difficulty that the dimensions are orthogonal. It is hard to see why the participant should not feel more positive after the pleasant words and more negative after the unpleasant words when the participant has the option to do so. Why feel more or less potent, when one can feel more or less pleasant as a result of the unconscious influence? One could argue that the conscious affective dynamics of valence are less sensitive to unconscious stimuli than affective dynamics of potency, but in the absence of any coherent theory of how affective dynamics actually operate and relate to semantic neural states, this would have to be a new working hypothesis as opposed to an explanation of the observed effects.

There is another line of explanation that is more speculative, but nevertheless worthy of consideration. First, our prior work suggests that the effects of stringently subliminal stimuli are generally *not* directly mirrored in consciousness (*i.e.*, do not produce main effects on measures that are isomorphic—here, the valence mood ratings), but rather yield bidirectional facilitative *vs.* inhibitory effects moderated by individual differences [5,23]. Such effects will be missed entirely unless revealed by relevant moderator analyses, and could explain the absence of valence mood main effects here. Similarly, Freud in *The Interpretation of Dreams* [36] posits that dream work transforms the affect in the unconscious latent dream into their opposite in the conscious dream—perhaps reflecting the inhibitory half of Snodgrass *et al.*’s bidirectional effects. Others (presumably those less threatened by the relevant unconscious material) might allow direct mirroring into conscious states. Without corroborating evidence of such bidirectional valence effects here, however, this possible explanation must remain tentative. 

But what about the potency effect? It may reflect an associative effect—but more in the Freudian, primary process sense (e.g., metaphor, analogy, concreteness, *etc.*), rather than standard “straightforward” cognitive associations as in typical priming paradigms (e.g., “reasonable” semantic associations). Such primary process associations may be more common under stringently subliminal conditions and/or when relevant motivational states are activated. For example, various New Look research from the 1940s and 1950s, more recently re-examined favorably by Erdelyi [37], suggests that in states of need (e.g., hunger) participants drew the diameter of a quarter larger than its true size. Presumably the state of need rendered the quarter more valuable or important—both nonperceptual factors. However, the effect of the greater hunger was not solely to *think* of the quarter as more important or valuable, but to alter the *perception* of the quarter so that it *looked* larger. Similarly, Finn *et al.* [38] have found that spider and snake phobics draw significantly larger versions of their phobic objects than other, nonphobic subjects do. Along the same lines, we have previously shown that unconscious processing of a stimulus is more subject to nonperceptual influences in the perceptual processing itself, not solely in accompanying thoughts [39]. Effects such as these are analogous to recent work top-down effects on perception in which attention, expectations, and past experience change the nature of the percept [40]. Notably, the Winkielman and Berridge experiment is an effort in this same direction. 

Given these considerations, here, we speculate that when the pleasant words stir up unconscious pleasant affect, this may be associatively reflected consciously as feeling relatively “bigger” (*i.e.*, more dominant, powerful), on the pictorial potency manikan scale, wherein subjects literally rate their feelings on a bigger *vs.* smaller scale. For unpleasant words, the reverse occurs, resulting in ratings of feeling “smaller” (*i.e.*, less dominant or powerful). At the same time, straightforward valence-related effects (*i.e.*, on the pleasant *vs.* unpleasant scale) are not directly mirrored in consciousness, as suggested above.

#### 2.1.2. Why Does This Conscious Potency Mood Effect Occur Only When There Is a Change from One Valence to Its Opposite in the Subliminal Stimuli?

The potency effect was surprisingly complicated. Conscious potency mood was not simply a function of whether the subliminal stimuli emotion-words were positive or negative, but whether they *changed* from one valence to its opposite. Further, as mentioned above, further analyses indicated that this effect then essentially disappeared by the last block, suggesting some kind of adaptation or “recovery” from the immediately preceding “valence change” effect. Overall, this pattern suggests an explanation in terms of *hedonic adaptation* [41]. Hedonic adaption/“set point” theory arose from the observation that the effects of many positive or negative events (excepting perhaps the most serious ones with lasting consequences) on mood was surprisingly short-lived, with a later return to baseline. Such adaption might be occurring completely unconsciously, with resultant shifts in conscious potency. Alternatively, conceivably the conscious mood change itself could trigger the adaptation process.

#### 2.1.3. Implications for the Existence of Unconscious Affect

The results of the present study pose a puzzling question: Which position on affect do they support? On the one hand, those who maintain that affect is always conscious can draw comfort from the result that there is in fact a conscious mood change associated with the presentation of completely subliminal emotion-word stimuli. On the other hand, those who maintain that affects can be unconscious could cite the same result by calling attention to the fact that it is not the *same* affect that was elicited consciously but rather an affect associatively but not directly related to valence—namely, potency. Thus, a subliminal affect valence could have registered and was associated with a conscious potency mood change. The result could then be understood as the registration of two different affects: an unconscious valence affect and a conscious potency affect. Both sides can claim support for their position.

It thus remains unclear whether subliminal activation induces unconscious affect or not. Is it a truly unconscious affect as Damasio [18], Berridge and Winkielman [19], and Shevrin [20] argue, or simply a state of unconscious *instigation* of a conscious affect, itself lacking affective qualities as Zajonc claims, or is it conscious in an affective state domain that is very different from the valence domain of cognitive information processing, as Panksepp [15] hypothesizes? Our results suggest that the choice among these alternatives may not be easy. Clearly something is unconscious and that something is categorically different, at least in the way we currently conceptualize affective categories, from the effect it has consciously on mood. It could be argued that in the present study what is unconscious is more cognitive than emotional. We have presented *words* subliminally, and even though they are affect words they are not the powerful stimuli that ordinarily provoke affects. Yet it is also the case that words alone on a printed page can elicit powerful affects, for example, in the form of novels, poems, and short stories. Certainly angry and happy faces are closer to the mark, but here we run into the problem of how unconscious they really can be made to be [30].

But why would pleasant and unpleasant words produce a change in conscious *mood* if they were simply cognitive in their impact? And why would they have an influence different from their actual connotation? The mood rating scales used were expressly chosen to be non-verbal and concrete, cartoon-like depictions of affects, rather than purely verbal accounts or descriptions of affect. Something like an affect must have been occurring unconsciously, or so it seems. Supporting this interpretation is evidence that electromyographic corrugator tension [42] and skin conductance responses to happy and angry schematic faces presented at the objective identification threshold are greater to angry faces [43]. Both facial emotional expressions and skin conductance responses are activated subcortically. SCR changes, for example, are caused by sympathetic innervation of exocrine sweat glands in the skin. The innervating neurons originate subcortically and thus would appear to involve the kind of subcortical activations Panksepp posits are involved in the deeper core affects. However, neither study measured concomitant conscious mood, thus failing to meet one of the two crucial criteria. To sustain the interpretation that an affect was unconscious in our studies, one would need to further assume that had we measured skin conductance and corrugator muscle tension it would have replicated the Bunce *et al.* [42] and Wong *et al.* [43] results.

Another possible problem remains in the way of concluding that interaction results support the existence of unconscious affects. One could argue that an essential control is lacking: What would happen if the same affect words were presented supraliminally? Might one find that the potency scale reflected the major shifts rather than the valence scale? Various evidence suggests this is unlikely. First, these stimulus words were taken from word norms reflecting the pleasant *vs.* unpleasant extremes of the Osgood scale, and hence clearly reflected strong valence differences. Further, when consciously presented, such stimuli have been repeatedly found to produce effects, albeit short-lived, on mood valence [44,45]. Yet there is no sign of such effects here, where the stimuli are rigorously subliminal.

There is another explanation that draws upon Panksepp’s hypothesis on the function of affect. Panksepp argues that affects must be conscious because only then can they perform their one important function: To *evaluate*—that is, assign value (e.g., good-bad, threatening-safe) to our perceptions. Our results might be interpreted along the same lines. The affect involved, pleasant or unpleasant, assigns value to the valence experience along a potency dimension. However, this interpretation requires that the unconscious stimuli activate a true unconscious affect, because according to Panksepp only affects perform this evaluative function. If, on the other hand, one argues in favor of a purely cognitive explanation of the subliminal valence stimuli, then one is hard put to explain why the resultant conscious mood does not simply mirror the subliminal valence manipulation—that is, simply produce conscious valence effects. In contrast, the primary process associative/metaphoric explanation above can—but likely then also implies unconscious evaluation, a function Panksepp reserves for conscious affect. Alternatively, Panksepp must accept the possibility that affects are not the only function that provides evaluations of ongoing experience.

#### 2.1.4. How Can Unconscious Affect Be Inferred?

Freud and James assumed that affect qualia could only be conscious, as have others more recently. This position, however, is simply definitional: If there is no conscious affect qualia, there can be no affect at all. In contrast, if unconscious affect is to be investigated scientifically, it must be testable, and proponents *vs.* skeptics must make falsifiable hypotheses. How might this be accomplished?

Discerning the presence of conscious affects is relatively straightforward—we just ask the subject. Since this is not possible with unconscious affects, they can only be indirectly inferred. Fortunately, when affects are stirred up, they are usually accompanied by various other processes. Indeed, when we speak of an affect or emotion we have in mind a number of facets that usually go together: physiological activation, expressive features registered in facial and gestural changes, cognitive content, and lastly, specific qualia or feeling state. Indeed, there is considerable variability in the way such words are used in the literature. Accordingly let us refer to the presence of all of these factors as an *emotional state*, and let us refer to the specific emotional feeling qualia as an emotional *affect*. This last factor is what is primarily at issue, and many would agree that affect is likely when the relevant emotional state can be observed.

As foreshadowed in the introduction, then, one approach would be to fulfill *all three* requirements in the same experiment—stringent subliminality, obtaining physiological markers of emotion, and the lack of at least the directly corresponding conscious affect (recalling that consciously presented stimuli *do* produce the corresponding conscious affect). For many, this would constitute reasonably compelling evidence for unconscious affects. Even here, however, skeptics could perhaps still maintain that the emotional state markers were somehow being caused purely cognitively, without the relevant affect qualia being present.

Perhaps the most powerful approach, then, would be to target the neurophysiological instantiation of affect qualia themselves—that is, the emotional brain areas that actually cause the emotional state markers. Notably, there is good evidence that there are many subcortical brain areas that are activated during strong emotional states in humans, and that some are common to many distinct states. For instance, Damasio *et al.* [46] have demonstrated that when people induce powerful affective states by vivid recollection, PET reveals that different subcortical areas including the periaqueductal grey (PAG) are activated depending on the emotion recollected. The emotions studied were sadness, happiness, anger, and fear. Panksepp [14,15] has argued that these areas of the PAG are highly active in all extreme basic emotional states. The PAG, then, may be just the neurophysiological instantiation of affect qualia desired, perhaps enabling an empirical settling of the controversy. Suppose we were to find that subliminal presentation of personally powerful emotional stimuli produced changes in emotional state markers consonant with the nature of the stimuli (e.g., autonomic arousal, facial expressions, cognitive discrimination), but no change in the corresponding conscious mood, *and with activation of the appropriate areas of the PAG*. Would we then be prepared to say that this is evidence for an unconscious affect? Or, supposing we found that everything was present marking an emotional state but PAG activation was lacking. Here, we might conclude either that the emotional marker activity was caused purely cognitively or that the PAG does not instantiate affect after all, and perhaps subsequently focus on other candidate brain areas (e.g., the amygdala, insula, *etc.*). Lastly, suppose that we found that in the presence of both PAG activation and the relevant emotional markers that the conscious mood reflected a *different* unrelated affect qualia, as in the studies reported in this paper. We might then be prepared to say that an unconscious emotional state exists along with its appropriate affect qualia, but that this state *interacts* with conscious qualia in ways not yet clearly understood, but perhaps consistent with Freud’s ideas relating to primary process associative properties. Alternatively, skeptics could still maintain that this unrelated conscious emotion was still somehow being caused purely cognitively.

We are thus led by the seemingly intractable nature of the controversy to imagine a thought experiment that would go beyond any experiment yet done, a daunting challenge. Of necessity it would need to be a PET study as in Damasio *et al.* [46] research, because PET is uniquely suitable for imaging slowly changing brain functions as well as the deeper, midline brain stem structures where the PAG is located. Equally important it requires using personally meaningful emotional stimuli comparable to those studied by Damasio *et al.* [46], but presented at the objective detection threshold. Along these lines, Shevrin *et al.* [47,48] have conducted ERP studies with personally meaningful verbal stimuli selected to reflect the unconscious causes of social phobias. However, the design requires a lengthy clinical evaluation and a complex word selection process. More recently, Aviyente *et al.* [49] have reported ERP differences to spider stimuli presented to spider phobics at the objective detection threshold. It would seem self-evident that to a phobic person the phobic object packs an emotional charge sufficient to result in all the accompaniments of a powerful emotional state including the affect qualia of fear. It would thus be appealing to have severe specific phobics as participants, both spider and snake phobics, for example, who would be exposed in a PET procedure to the relevant phobic stimuli presented at the objective detection threshold. It would also be required that conscious mood be measured as well as skin conductance and changes in facial musculature. Perhaps in this kind of experiment the questions raised might be answered one way or the other, and the issue of unconscious affect achieve some modicum of resolution.

Of course, a skeptic might respond either that the PAG does not instantiate affect after all, or perhaps again claim that it merely causes conscious affect rather than reflects actual unconscious affects (if, as here, there was evidence for some effects on conscious mood, albeit not directly corresponding ones). Ultimately, however, unless critics of unconscious affect are prepared to accept *some* brain instantiation of affect qualia (or other emotional markers caused by such brain areas, such as SCR), such skeptical positions become untestable in principle and hence outside scientific inquiry altogether.

## 3. The Study

### 3.1. Description of Experiments

Two experiments were conducted, the second an exact replication of the first. Participants were told that we were interested in spontaneous mood changes, which occur quite often in the course of a day. About every ten minutes they would be asked to rate their moods on several scales. In between the mood ratings they were told that we would be conducting another experiment on perception in which they would be asked to judge whether very quick flashes presented in a tachistoscope were bright or dim. In fact, no flash or change in illumination occurred because both the stimulus and fixation fields were matched for luminance. Thus there was no hint of any stimulus possible, which was confirmed in the subsequent threshold detection procedure.

### 3.2. Participants

Participants were recruited by a newspaper ad in the undergraduate and the local city newspapers. They were men and women between the ages of 18 and 55. English had to be their first language and their eyesight had to be corrected to at least 20/30. They were paid 20 dollars for an experiment lasting approximately an hour and a half. There were 27 participants in the first study and 28 in the second study.

### 3.3. Stimuli

The subliminal stimuli were twenty words drawn from the pleasant and unpleasant extremes of Osgood’s valence dimension [31], ten pleasant and ten unpleasant (see Table 3). These same words had been used in the Bernat *et al.* [29] study in which the subliminal unpleasant words compared to the subliminal pleasant words were associated with more positive ERP amplitude. The stimuli were printed on 4 × 6 cards in a Helvetica Light 18 font. They were presented in a three-field tachistoscope that made possible 1msec duration. Luminance was set at 5 foot/lamberts in the tachistoscope as well as in the sound proofed booth in which the experiment was conducted. These were the same conditions used in Bernat *et al.* [29]. 

**Table 3:** Pleasant and unpleasant words.

Pleasant Words	Unpleasant Words
Loving	Angry
Affectionate	Hostile
Tranquil	Envious
Elated	Unhappy
Relaxed	Sad
Excited	Jealous
Calm	Depressed
Enthusiastic	Distressed
At rest	Fearful
Warm-hearted	Agitated

### 3.4. Mood Measurement

Mood was measured with the manikin valence, potency and arousal scales [34]. The non-verbal manikin scales were selected for two reasons: (1) The manikin scales have been fully validated in a number of studies (see [50] for a review), and (2) since the subliminal stimuli were words it was thought that verbal mood measures might simply measure semantic relationships between the two sets of words rather than mood. Participants were asked to make a mark along a Visual Analog Scale [51] immediately below the manikin. This allowed for a more refined measure of mood than to have participants make a mark either immediately below the middle of each manikin or between the manikins as is usually done. The length of the line marked was 13 cm.

After giving the initial instructions, participants made a baseline mood rating on the three manikin scales. After a block of 10 words were presented participants made another mood rating, following which the same block of 10 words were presented and another mood rating collected. The next two blocks were from the opposite valence and two more mood ratings were obtained. In all, the 10 valence words, pleasant or unpleasant, were presented twice each and five (one baseline, four experimental) mood ratings obtained. 

### 3.5. Detection Procedure

After the subliminal presentations and mood ratings were concluded, a detection series was administered in which all of the words were presented plus an equal number of blanks in random order. Participants responded by saying whether after each presentation they saw a word or a blank. 

## 4. Conclusion

The results of our two experiments it must be concluded are still subject to differing interpretations whose resolution awaits the kind of experiment described above. At the same time the research design and results of the present experiments clarify the issues by including conscious mood measurements linked to verbal stimuli presented at the objective detection threshold, thus correcting the limitations of previous studies. However, in view of the continued differences in interpretation of the results among the authors, we conclude that conscious affective changes can be provoked by completely unconscious presentations of verbal stimuli directly linked to affects that are orthogonal to the conscious affect changes. This intriguing finding, however, neither rules in nor rules out the existence of unconscious affect. The resolution of this quandary would require more sophisticated experimental designs and measurements, perhaps like the PET study described previously.

## References

[1] Bernat E., Shevrin H., Snodgrass M. (2001). Subliminal visual oddball stimuli evoke a P300 component. Clin. Neurophysiol..

[2] Greenwald A.G., Draine S.C., Abrams R.L. (1996). Three cognitive markers of unconscious semantic activation. Science.

[3] Merikle P.M., Smilek D., Eastwood J.D. (2001). Perception without awareness: perspectives from cognitive psychology. Cognition.

[4] Shevrin H., Fritzler D.E. (1968). Visual evoked response correlates of unconscious mental processes. Science.

[5] Snodgrass M., Bernat E., Shevrin H. (2004). Unconscious perception: A model-based approach to method and evidence. Percept. Psychophys..

[6] Snodgrass M., Shevrin H., Kopka M. (1993). The mediation of intentional judgments by unconscious perceptions—The influences of task strategy, task preference, word meaning, and motivation. Conscious. Cogn..

[7] Freud S., Strachey J. (1953). The Unconscious. The Standard Edition of the Complete Psychological Works of Sigmund Freud.

[8] James W. (1890). Principles of Psychology.

[9] Clore G.L., Ekman P., Davidson R.J. (1994). Why emotions are never unconscious. The Nature of Emotion: Fundamental Questions.

[10] Lambie J.A., Marcel A.J. (2002). Consciousness and the varieties of emotion experience: A theoretical framework. Psychol. Rev..

[11] Zajonc R.B., Forgas J.P. (2000). Feeling and thinking: Closing the debate over the independence of affect. Feeling and Thinking: The Role of Affect in Social Cognition.

[12] Zajonc R.B., Gilbert D.T., Fiske S.T., Lindzey G. (1998). Emotions. The Handbook of Social Psychology.

[13] Breuer J., Freud S., Strachey J. (1955). Studies on hysteria. The Standard Edition of the Complete Psychological Works of Sigmund Freud.

[14] Panksepp J. (1998). Affective Neuroscience: The Foundations of Human and Animal Emotions.

[15] Panksepp J. (2003). Can anthropomorphic analyses of separation cries in other animals inform us about the emotional nature of social loss in humans? Comment on Blumberg and Sokoloff (2001). Psychol. Rev..

[16] Panksepp J. (2005). Why does separation distress hurt? Comment on MacDonald and Leary (2005). Psychol. Bull..

[17] Panksepp J. (2005). Affective consciousness: Core emotional feelings in animals and humans. Conscious. Cogn..

[18] Damasio A.R. (1999). The Feeling of What Happens: Body and Emotion in the Making of Consciousness.

[19] Berridge K.C., Winkielman P. (2003). What is an unconscious emotion? (The case for unconscious “liking”). Cogn. Emot..

[20] Shevrin H., Velmans M. (2000). The experimental investigation of unconscious conflict, unconscious affect, and unconscious signal anxiety. Investigating Phenomenal Consciousness: New Methodologies and Maps.

[21] Ohman A., Flykt A., Lundqvist D., Lane R.D. (2000). Unconscious emotion: Evolutionary perspectives, psychophysiological data and neuropsychological mechanisms. Cognitive Neuroscience of Emotion.

[22] Reingold E.M., Merikle P.M. (1990). On the inter-relatedness of theory and measurement in the study of unconscious processes. Mind Lang..

[23] Snodgrass M., Shevrin H. (2006). Unconscious inhibition and facilitation at the objective detection threshold: Replicable and qualitatively different unconscious perceptual effects. Cognition.

[24] Macmillan J.F., Gold A., Crow T.J., Johnson A.L., Johnstone E.C. (1986). The Northwick Park study of 1st episodes of schizophrenia: IV. Expressed emotion and relapse. Br. J. Psychiatry.

[25] MacMillan J.F., Creelman C.D. (1991). Detection Theory: A User’s Guide.

[26] Erdelyi M.H. (1986). Psychoanalysis has a wider scope than the retrospective discovery of etiologies. Behav. Brain Sci..

[27] Holender D. (1986). Semantic activation without conscious identification in dichotic-listening, parafoveal vision, and visual masking: A survey and appraisal. Behav. Brain Sci..

[28] Snodgrass M. (2002). Disambiguating conscious and unconscious influences: Do exclusion paradigms demonstrate unconscious perception?. Am. J. Psychol..

[29] Bernat E., Bunce S., Shevrin H. (2001). Event-related brain potentials differentiate positive and negative mood adjectives during both supraliminal and subliminal visual processing. Int. J. Psychophysiol..

[30] Winkielman P., Berridge K.C., Wilbarger J.L. (2005). Unconscious affective reactions to masked happyversus angry faces influence consumption behavior and judgments of value. Pers. Soc. Psychol. Bull..

[31] Osgood C.E., Suci G.J., Tannenbaum P.H. (1957). The Measurement of Meaning.

[32] Winkielman P., Zajonc R.B., Schwarz N. (1997). Subliminal affective priming resists attributional interventions. Cogn. Emot..

[33] Murphy S.T., Zajonc R.B. (1993). Affect, cognition, and awareness: Affective priming with optimal and suboptimal stimulus exposures. J. Pers. Soc. Psychol..

[34] Bradley M.M., Lang P.J. (1994). Measuring emotion: The self-assessment mannequin and the semantic differential. J. Behav. Ther. Exp. Psychiatry.

[35] Kunst-Wilson W.R., Zajonc R.B. (1980). Affective discrimination of stimuli that cannot be recognized. Science.

[36] Freud S., Strachey J. (1953). The interpretation of dreams. The Standard Edition of the Complete Psychological Works of Sigmund Freud.

[37] Erdelyi M. (1974). A new look at the new look: Perceptual defense and vigilance. Psychol. Rev..

[38] Finn M., Shevrin H., Brakel L., Snodgrass M. (2012). Specific phobics judge their phobic objects to be larger than nonphobics.

[39] Shevrin H. (1973). Brain wave correlates of subliminal stimulation, unconscious attention, primary- and secondary-process thinking and repression. Psychol. Issues.

[40] Gilbert C., Sigman M. (2007). Top-down influences in sensory processing. Neuron.

[41] Frederick S., Loewenstein G., Kahneman D., Diener E., Schwartz N. (1999). Hedonic adaptation. Well-Being: The Foundations of Hedonic Psychology.

[42] Bunce S.C., Bernat E., Wong P.S., Shevrin H. (1999). Further evidence for unconscious learning: preliminary support for the conditioning of facial EMG to subliminal stimuli. J. Psychiatr. Res..

[43] Wong P.S., Shevrin H., Williams W.J. (1994). Conscious and nonconscious processes: An ERP index of an anticipatory response in a conditioning paradigm using visually masked stimuli. Psychophysiology.

[44] Barrett L., Ochsner K., Gross J., Bargh J. (2007). On the automaticity of emotion. Social Psychology and the Unconscious: The Automaticity of Higher Mental Processes.

[45] Topolinsky S., Deutsch R. (2012). Phasic affective modulation of semantic priming. J. Exp. Psychol. Learn. Mem. Cogn..

[46] Damasio A.R., Grabowski T.J., Bechara A., Damasio H., Ponto L.L., Parvizi J., Hichwa R.D. (2000). Subcortical and cortical brain activity during the feeling of self-generated emotions. Nat. Neurosci..

[47] Shevrin H. (1996). Conscious and Unconscious Processes: Psychodynamic, Cognitive, and Neurophysiological Convergences.

[48] Shevrin H., Williams W.J., Marshall R.E., Hertel R.K., Bond J.A., Brakel L.A.W. (1992). Event-related potential indicators of the dynamic unconscious. Conscious. Cogn..

[49] Aviyente S., Brakel L.A.W., Kushwaha R.K., Snodgrass M., Shevrin H., Williams W.J. (2004). Characterization of event related potentials using information theoretic distance measures. IEEE Trans. Biomed. Eng..

[50] Bradley M.M., Lang P.J., Lang P.J., Nadel L. (2000). Measuring emotion: Behavior, feeling, and physiology. Cognitive Neuroscience of Emotion.

[51] Gift A.G. (1989). Visual analog scales: Measurement of subjective phenomena. Nurs. Res..

